# EMT and Stemness—Key Players in Pancreatic Cancer Stem Cells

**DOI:** 10.3390/cancers11081136

**Published:** 2019-08-08

**Authors:** Eva Rodriguez-Aznar, Lisa Wiesmüller, Bruno Sainz, Patrick C. Hermann

**Affiliations:** 1 Department of Internal Medicine I, Ulm University, 89081 Ulm, Germany; 2 Division of Gynecological Oncology, Department of Obstetrics and Gynecology of the University of Ulm, 89081 Ulm, Germany; 3 Department of Biochemistry, Universidad Autonoma de Madrid (UAM), 28029 Madrid, Spain; 4 Department of Cancer Biology, Instituto de Investigaciones Biomedicas “Alberto Sols” (IIBM), CSIC-UAM, 28029 Madrid, Spain; 5 Chronic Diseases and Cancer, Area 3-Instituto Ramon y Cajal de Investigacion Sanitaria (IRYCIS), 28029 Madrid, Spain

**Keywords:** cancer stem cells, pancreatic cancer, metastasis, EMT, stem cells

## Abstract

Metastasis and tumor progression are the major cause of death in patients suffering from pancreatic ductal adenocarcinoma. Tumor growth and especially dissemination are typically associated with activation of an epithelial-to-mesenchymal transition (EMT) program. This phenotypic transition from an epithelial to a mesenchymal state promotes migration and survival both during development and in cancer progression. When re-activated in pathological contexts such as cancer, this type of developmental process confers additional stemness properties to specific subsets of cells. Cancer stem cells (CSCs) are a subpopulation of cancer cells with stem-like features that are responsible for the propagation of the tumor as well as therapy resistance and cancer relapse, but also for circulating tumor cell release and metastasis. In support of this concept, EMT transcription factors generate cells with stem cell properties and mediate chemoresistance. However, their role in pancreatic ductal adenocarcinoma metastasis remains controversial. As such, a better characterization of CSC populations will be crucial in future development of therapies targeting these cells. In this review, we will discuss the latest updates on the mechanisms common to pancreas development and CSC-mediated tumor progression.

## 1. Pancreatic Development

**Figure 1:**
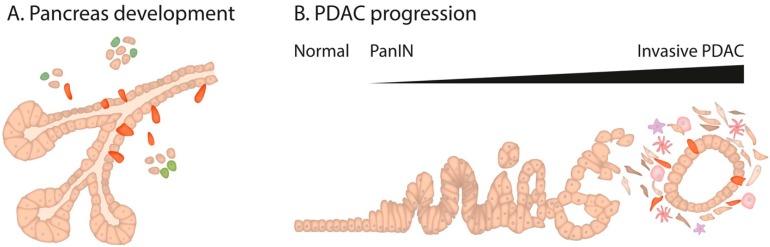
The epithelial-to-mesenchymal transition (EMT) process during pancreatic development and pancreatic ductal adenocarcinoma (PDAC) progression. During embryonic pancreas development, differentiation of endocrine cells occurs along the secondary transition: Cells within the epithelium upregulate Ngn3 and delaminate in a partial EMT-like process (red) to coalesce and form the Islets of Langerhans (differentiated endocrine cells in green). PDAC progression from normal tissue to pancreatic intraepithelial neoplasia (PanIN) lesions to invasive PDAC. Cells undergoing EMT are able to delaminate from the epithelium and migrate. Cells in red depict cells with expression of EMT-TFs, either undergoing EMT or already present in the stroma.

The pancreas arises from the posterior foregut of the definitive endoderm [1] and first becomes evident around embryonic day E8.5 in mice and prior to 26 days of gestation in humans, with the emergence of dorsal and ventral buds, which fuse by E12.5 (Carnegie stage 18–20 in humans) [2,3]. Sonic hedgehog (Shh) repression from the mesenchyme and the notochord together with retinoic acid (RA), FGF and BMP signaling participate in the specification and expansion of the pancreatic domains [4,5]. During the primary transition, active cell proliferation and extensive branching morphogenesis expand the epithelium into the surrounding mesenchyme in a tree-like structure, increasing the size of the pancreatic buds [6] (Figure 1A). At this stage, the epithelium is characterized by the expression of the transcription factors Pdx1, Ptf1a, and Sox9 [7,8,9]. During the secondary transition (E12.5 until birth), the developing pancreas undergoes rapid morphological changes, tightly controlled by a milieu of systemic signals and signaling pathways through which undifferentiated multipotent progenitors commit to the endocrine, acinar or ductal lineages that are found in the mature pancreas. Notch signaling is essential in the spatiotemporal control of lineage allocation, segregating cells within the epithelium into acinar progenitors at the tip and bipotent progenitors at the trunk domains. Notch activity is implicated in the subsequent binary fate determination of the bipotent progenitors to differentiate into either ductal or endocrine progenitor cells. Low Notch levels allow Ngn3 to rise in scattered epithelial cells, which will delaminate into the mesenchyme to coalesce and form the future islets [10]. After birth, beta-cell development continues with islet cell expansion and maturation.

The adult pancreas is comprised of two functionally distinct compartments. On the one hand, the exocrine pancreas, which consists of acinar and ductal cells making up 96–99% of the total organ mass. On the other hand, the endocrine islets of Langerhans are comprised of five specialized cell types (α, β, δ, PP and ε cells), secrete various hormones (with insulin and glucagon being the most important ones) and are essential for the regulation of glucose metabolism. 

The mammalian pancreas displays a significant capacity for regeneration following injury, with the acinar compartment conserving the highest plasticity in both mouse and human. Through changes in transcription factor expression and epigenetic regulation, acinar cells are able to de-differentiate to an embryonic progenitor-like phenotype to commit to either beta cells or ductal cells, the latter case known as acinar-to-ductal metaplasia (ADM). ADM trans-differentiation occurs in chronic pancreatitis, and has been postulated to be a necessary step in the generation of neoplastic precursor lesions termed pancreatic intraepithelial neoplasia (PanINs) [11,12]. 

## 2. Pancreatic Cancer

Pancreatic ductal adenocarcinoma (PDAC), the most common form of pancreatic cancer, is projected to be the second most frequent cause of cancer-related death by 2030 [13]. It is more prevalent in males, and increasing age, smoking, obesity, diabetes, and chronic pancreatitis are contributing risk factors [14]. Unspecific symptoms usually delay diagnosis, which is further hampered by the lack of reliable biomarkers. As a consequence, most pancreatic cancers are diagnosed at an advanced stage of the disease, making less than 20% of the patients eligible for resection (and potential cure). Hence the majority of patients must undergo systemic therapy [15,16,17]. The five-year survival rate still remains low at approximately 9%. Currently, the most successful chemotherapy combinations are FOLFIRINOX (folic acid, 5-FU, irinotecan, and oxaliplatin) and gemcitabine plus nab-paclitaxel, prolonging patient survival by 6–12 months [18,19]. While many factors contribute to the failure of chemotherapy in PDAC, the extensive desmoplastic response in the tumor microenvironment (TME) plays an essential role, where a dense extracellular matrix and the presence of cancer-associated fibroblasts (CAFs), activated pancreatic stellate cells (PSCs), mesenchymal stem cells (MSCs) and immune cells (e.g., tumor-associated macrophages (TAMs)) [20,21] form a protective and supportive niche for tumor cells.

Significant advances in understanding pancreatic tumor biology have been made, revealing the complexity and heterogeneity of this disease. Large-scale genomic analyses have unraveled the mutational landscape of PDAC, leading to the identification of candidate pathways promoting tumorigenesis, many of which also operate during embryonic development [22]. Accumulating genetic alterations in KRAS, TP53, CDKN2A, and SMAD4 have been identified as major drivers during the progression from low-grade PanIN lesions to PDAC [23] (Figure 1B). KRAS oncogene mutation is widely accepted to represent an initiating event, supported by the finding that it is the most frequently (>90%) mutated gene in PDAC [22,24]. In fact, genetically engineered mouse models (GEMMs) with KRAS mutations in pancreatic progenitor cells recapitulate human PDAC tumorigenesis, progression, and metastasis. These animals are usually referred to as KC or KPC for expression of *LSL-Kras^G12D^* mutation and an *LSL-Trp53* or a *Trp53* mutation under the control of a Pdx1 or Ptf1a- driven Cre recombinase [25,26]. However, different molecular signatures have allowed the classification of PDAC into different subtypes, and to the proposal of a phylotranscriptomic tree [27]. Transcriptional modifications and epigenetic analyses seem to recapitulate two main phenotypes: The “classical” and the “basal” subtypes [28,29]. Nevertheless, these and other studies have only confirmed tumor complexity, emanating intrinsically from clonal subpopulations with varying molecular and functional properties [30,31] such as a highly plastic stem-like population found within the tumor, important for tumor initiation and progression. 

## 3. (Cancer) Stem Cells

Stem cells are undifferentiated cells mainly characterized by their unlimited capacity to proliferate, leading to both self-renewal and differentiation into different progenies from embryonic development (ESC) throughout adulthood (adult or somatic stem cells). ESCs derive from the blastocyst’s inner cell mass and are totipotent, i.e., they can generate cells of all (ectoderm, endoderm and mesoderm) cellular lineages of a developed organism. Adult stem cells are tissue-specific stem cells able to generate transit-amplifying progenitor cells that fully differentiate into the mature cells of the tissue in which they reside. 

Unlimited proliferation potential, self-renewal, and resistance to apoptosis are stem cell traits mirrored by cancer cells. Together with these characteristics, a cell must also acquire self-sufficiency in growth signals, insensitivity to growth-inhibitory signals, and increased cellular motility in order to become cancerous [32]. Tumor heterogeneity was initially thought to be the result of stochastic genetic and/or epigenetic mutations in individual cells, giving rise to a clonal progeny with a selective growth advantage. More recently, the strong similarities between cancer and embryonic development led to the hypothesis that a hierarchy exists within the tumor, with a unique population of cancer stem-like cells (also termed tumor-initiating cells) sustaining cancer progression [33,34].

Cancer stem cells (CSCs) were first identified in hematological cancers [35,36], followed by their detection in practically all solid tumors, including PDAC [37,38]. Defining features for CSCs are their tumor-initiating capacity, unlimited self-renewal, and ability to regenerate the cellular heterogeneity of the parental tumor after implantation into secondary recipients. Furthermore, CSCs have been described to be critically involved in metastatic dissemination and therapy resistance [37,39,40,41]. Although the precise cellular origin of CSCs remains unclear, the functional similarities with stem cells suggest that CSCs could arise from a transformed stem or progenitor cell, or through de-differentiation of differentiated cells present in adult tissues [42]. 

In the adult pancreas, even terminally differentiated cells show a high degree of plasticity, capable of adopting features of a different pancreatic lineage. Such is the case for acinar, alpha, and beta cells in particular, as demonstrated by their neogenesis, de-differentiation, and trans-differentiation potential following injury [43,44,45,46]. Cells expressing the neural stem cell-specific marker nestin were discovered within islets and pancreatic ducts could be expanded and differentiated in vitro, suggesting multipotency [47]. However, the existence of rare cells at the junction between acini and the adjacent ductal epithelium, which actively maintain developmental programs as shown by Notch activation and expression of PDX1, Ptf1a and Sox9, paved the way to propose that centro-acinar cells are the bona fide resident stem/progenitor cells in the adult pancreas [8,48,49]. These cells are characterized by a high nuclear-to-cytoplasmic ratio with long extensions, a fast proliferation response after partial pancreatectomy, streptozotocin (STZ) or caerulein administration, and the ability to generate different cell types [50,51,52]. They have been successfully isolated based on ALDH activity, which has been associated with stem or progenitor cells in different physiological contexts to maintain the progenitor status, as well as in different cancers including PDAC [53,54]. 

Additional properties inherent to CSCs are 26S proteasome activity [55], autofluorescence [56], expression of doublecortin like kinase 1 [57], FoxM1 [58], FAM83A [59], RORγ [60], and specific cell surface antigen and receptor expression such as CD44, CD90, CD24, CD133, EpCAM, LGR5, CXCR4 and c-Met [61]. Of these properties, cell surface markers represent the most common method for the isolation of CSCs. Interestingly, most of these surface markers are present on normal embryonic or adult stem cells. CD133 is expressed in adult pancreatic ducts, and a subpopulation of cells among CD133+ ductal cells has been shown to consist of progenitor cells with multilineage differentiation capacity that also express c-Met [62]. CD44 and CD133 are co-expressed in centroacinar cells in the normal adult pancreas [63]. CXCR4 is expressed during pancreatic development and is involved in cell migration during regeneration [64]. EpCAM is present in the epithelium of human fetal pancreas at 18–20 weeks of gestational age during endocrine cell development [65] and LGR5 is co-expressed with Nanog during embryonic development of the pancreas [66]. The list of CSC markers has grown far beyond surface markers and now includes miRNAS and long non-coding RNAs, differences in exosome composition [67,68] upregulation of proteins in the CSC secretome such as fatty acid synthase (FASN), acetoacetyl-CoA transferase (ACAT2), ceruloplasmin, galectin-3, MARCKS and CA19-9 [69,70,71], which might even serve as biomarkers However, conflicting data have arisen over the use of some of these, since their expression varies with different isolation and culture conditions [72]. Nevertheless, due to the lack of a universal “perfect” CSC marker with 100% sensitivity and specificity, all isolation methods to date have only resulted in (high) enrichment of CSCs within the respective target population. This has resulted in obligatory experimental and functional validation for the identification of CSCs, such as extreme limiting dilution assays (ELDA) to measure tumorigenicity in immunocompromised recipient mice [73]. Sphere or colony-forming assays are common methods used to assess the self-renewal and differentiation potential of putative stem cells to grow in non-adherent serum-free conditions and have been applied to CSC biology with tremendous success [39,74]. In this respect, spheres have also been successfully derived from normal pancreatic cells with ALDH activity that were also enriched for Sox9, Sca-1, c-Met and Nestin [75].

CSCs reactivate embryonic programs [76], and the expression of genes associated with self-renewal such as c-Myc, Oct4, Sox2, and Nanog is further common ground for stem cells and CSCs [77,78]. In order to maintain their proliferative capacity, stem cells overcome telomere attrition by maintaining telomerase activity. CSCs are also endowed with telomere length stabilization mechanisms in order to keep unlimited self-renewal leading to cellular immortalization [79]. Autophagy is an additional cellular process linked to stem cell self-renewal and differentiation and to the biology of CSCs [80,81]. Alterations in the metabolic state of the cells also drive stemness and tumor heterogeneity: Stem cells favor glycolysis since mitochondria are considered to be in an immature state resulting in low OXPHOS, ATP and ROS levels [82]. On the other hand, CSCs display high mitochondrial content, and CSCs are highly dependent on OXPHOS as compared to differentiated cells [83]. A hypoxic microenvironment maintaining an undifferentiated cell state during pancreas development has been observed [84], since hypoxia induces stemness via the upregulation of key pluripotency factors and additionally contributes to PDAC aggressiveness favoring invasion and metastasis [85,86].

An alternative hypothesis reconciles both the stochastic and the hierarchal CSC models, suggesting that phenotypic plasticity among cancer cells, shifting between CSC and non-CSC states, is responsible for the development and maintenance of cancer [87], and that “stemness” in cancer cells may be a state, rather than an entity [88]. Although most neoplasms arise from a single cell, accumulation of genetic variability over time results in the generation of multiple subclones [30]. Makohon-Moore et al. recently described the evolution of pancreatic cancer as a multi-step progression originating from a single mutant clone, which spreads through the pancreatic ductal system to generate additional neoplastic lesions [89]. Multi-color confetti lineage tracing studies in mice showed that distinct lesions originated from independent ADMs, consistent with the presence of polyclonal metastatic seeding. However, PanINs rapidly become monoclonal, confirming clonal selection as an early event in tumor progression [90]. Using PCR-based integration site amplification, tumor formation from patient-derived PDAC could be traced to a few cells, which were found to be functionally distinct in subsequent serial xenotransplantions, implying a succession of tumor-initiating cells (TICs) over time with high plasticity in PDAC xenografts [91]. Seth et al. recently generated clonal replica tumors from patient-derived pancreatic cancer. In this case, tumors generated from xenotransplanted cells showed rapid clonal exhaustion, with only a small population of TICs endowed with long-term self-renewal, sustaining pancreatic tumor growth during in vitro and in vivo passaging [92]. These apparent discrepancies could be attributed to the fact that clones initially arise with a growth advantage, then diversify, evolve and adapt in response to changes such as local factors in the TME or (chemo) therapy, which could result in the selection and expansion of clones underrepresented initially [93]. 

Recent studies have identified the epithelial-to-mesenchymal (EMT) transition as a key mechanism by which cells are conferred with stem-cell properties. Together with stemness, plasticity, invasion, and chemoresistance are all features attributed to EMT as we will discuss below.

## 4. EMT in Pancreatic Development and Cancer

An epithelial-to-mesenchymal transition was first described as a process during which an epithelial cell acquires migratory properties, characterized by the repression of epithelial markers and the appearance of mesenchymal attributes [94,95].

Although EMT was first observed in embryonic development during mesoderm formation and in neural crest delamination, it soon became evident that it comprises an evolutionarily conserved mechanism that fulfills important roles in vertebrate development: EMT transcription factors (TFs) are required for several cell movements, with which (under several conversions of EMT and the reverse process (MET) organisms change their shape during development [96]. In order to delaminate and colonize distant organs, cells undergoing EMT not only acquire the capacity to migrate, but also undergo numerous changes together with cell cycle exit and resistance to apoptosis. These changes are mainly orchestrated by transcriptional repressors including members of the Snail, Zeb, Twist, and Prrx families, and are fine-tuned by the action of several miRNAs [97].

Several lines of evidence support the notion that delamination of pancreatic endocrine cells from the developing epithelium is a process reminiscent of EMT. Ngn3 expressing cells co-express the epithelial marker E-cadherin and the mesenchymal marker vimentin [98], suggesting that an intermediate or partial EMT governs endocrine delamination from the developing epithelium. Snai1 and Snai2 transcripts have been detected in both the epithelium and the mesenchyme of the developing pancreas [99]. Microarray analysis of DBA isolated ductal cells depicted the expression of Prrx1 in embryonic pancreata [100]. Single-cell RNA-sequencing of cells encompassing different stages of Ngn3 positive cell differentiation determined the step-wise trajectory of endocrine progenitor maturation, with delaminating cells clearly enriched in EMT signature markers including Snai1, Snai2, and Twist [101]. More specifically, at E15.5, Snai2 is strongly co-expressed with Ngn3 which, in turn, promotes endocrine precursor cell delamination through Snai2 expression [99,102]. The transcriptional coactivator YAP1, which has been implicated in several developmental processes including EMT and tumorigenesis [103], is also expressed in the developing pancreas during the secondary transition, regulating the correct specification of endocrine cells [104]. 

EMT can be reactivated in the adult as a response to stimuli such as injury, or also during organ fibrosis and cancer progression [96]. In the adult pancreas, Snai2 expression becomes restricted to the islets of Langerhans [102], whereas Snai1 expression is confined to cells mainly described as pancreatic mesenchymal cells [105]. Prrx1 remains present in some ductal cells but is expressed at much lower levels than during embryonic development [100]. It has been proposed that EMT could account for the de-differentiation of isolated human islet cells into cells of a mesenchymal phenotype, which could expand and then revert back to a population of insulin-producing cells. However, more experiments are necessary to clarify the origin of these transit-amplifying cells [106,107].

Despite limited knowledge regarding the expression and function of EMT TFs during normal pancreas development under physiological conditions, a plethora of EMT-mediating TFs are known to be re-activated in cancer, and an “EMT cancer signature” comprising 16 key genes has been compiled [108]. Many of these are regulated through the activation of signaling pathways in neoplastic cells, such as TGFb, WNTs, Notch and mitogenic growth factors [109]. The phenotypic changes associated with EMT contribute to the appearance of CSCs, plasticity, delamination, CTC generation, metastasis formation, and therapy resistance.

## 5. EMT and CSCs

EMT, initially considered a switch for epithelial cells to assume a mesenchymal state, is a step-wise process covering a spectrum of intermediate “meta-stable” phases [97]. Multiple studies have attempted to ascertain the existence of these intermediary phenotypes. For example, based on a screening for a large panel of cell surface markers, different combinations of EpCAM, CD51, CD61, and CD106 discriminate six distinct populations comparable to different EMT transition states in squamous cell carcinoma [110]. Furthermore, this study shows that tumor cells with hybrid epithelial and mesenchymal traits are more efficient in reaching the circulation, colonizing the lungs and forming metastases, substantiating previous findings linking these intermediate states with the acquisition of stemness features.

The notion that cells undergoing EMT acquire enhanced mesenchymal and stemness traits first came from work on the mammary gland and breast cancer cells [111,112]: Here, cooperation between the transcription factors Snai2 and Sox9 is necessary to determine a mammary stem cell fate [113]. Furthermore, sequential EMT-MET steps mediating epithelial plasticity have been shown to be crucial during OKSM-mediated reprogramming of murine embryonic fibroblasts (MEFs) into iPSCs [114,115,116].

The link between EMT activation and CSCs has been extensively recognized in a variety of human carcinomas [117]. Using different murine PDAC and pancreatitis models, Rhim et al. demonstrated that cells which have undergone a partial EMT and express E-cadherin and Zeb1 exhibit stem cell properties [118]. Zeb1 reactivation promotes EMT together with the expression of the pluripotency factors Sox2 and Klf4 [119]. Furthermore, KPC murine pancreatic tumor cells devoid of Zeb1 are deficient in stemness and colonization properties [120]. CD133, a surface antigen associated with CSCs in human PDAC, mediates EMT through the regulation of Snai2 in human pancreatic cells [121]. Human PDAC sphere cultures enrich for CSC markers such as CD44, Aldh1a1, and Nanog, and also show increased Snai1 expression levels [122]. In the pancreas, Prrx1+ cells were more efficient in sphere formation and, when animals were subjected to caerulein-induced pancreatitis, increased numbers of Prrx1+ cells were found, and the Prrx1b isoform specifically drove Sox9 expression [100]. Moreover, in different PDAC models, this isoform promotes invasion and EMT, whereas Prrx1a was found to be more prominent in metastasis, suggesting that isoform switching can regulate EMT states [123]. Furthermore, downregulation of Prrx1 in human breast cancer cells was associated with MET, together with induced sphere formation and gain of CD44 [124]. Interestingly, metastatic lesions exhibit increased epithelial characteristics, resembling the initial parental tumor, as they grow [125].

Stromal cells support and promote the pancreatic CSC population via multiple signaling pathways [126]. Molecules secreted from the microenvironment, such as TGFb, IL-6, EGF, VEGF, and HGF support CSC formation, survival and EMT [109]. hCAP-18/LL-37 and ISG15 secreted by TAMs and LIF secreted by stellate cells promote stemness, concomitant with the activation of an EMT program and enhanced migration and invasion [127,128]. Indeed, the role of TAMs in activating the CSC compartment is an ever-increasing field of study [129].

## 6. EMT, CTCs and Metastasis

Since CSCs are essential for metastatic spread [37], they might be the population that ultimately gives rise to circulating tumor cells if the CSC hypothesis holds true. Indeed, increased aggressiveness of tumors has been related to EMT and elevated numbers of CTCs [130], and CTCs have been demonstrated to have both tumor-initiating and EMT traits [131], suggesting that these populations are indeed closely related. Furthermore, most CTCs do not proliferate and resistant to chemotherapy [132]. The metastatic process involves a cascade of steps from invasion to metastasis, from intravasation to extravasation and colonization of a distant organ [133]. Cells that dissociate from the primary tumor and enter the circulation, i.e., CTCs, are considered to be the founders of metastatic colonization.

As early as 1869 cells resembling the cancer cells were discovered in the blood upon autopsy [134]. Since then, our understanding of metastasis as a complex process has improved a lot, but at the same time advances in technology have enabled researchers to approach the topic of CTCs with considerably better equipment. In a landmark paper on CTCs, Cristofanilli et al. demonstrated for metastatic breast cancer that the detection of as few as five CTCs in a standardized system (CellSearch, Veridex) was sufficient to independently predict poor outcome [135]. Interestingly, the relevance of CTCs as a prognostic factor was confirmed in breast cancer also for the time of primary diagnosis [136]. The detection of CTCs can be successfully performed using the CellSearch system in virtually all solid tumors with reasonably high detection rates [137]. While since the introduction of the CellSearch system, various detection methods have been used successfully (e.g., FACS, microfluidics), CellSearch remains the most commonly used system due to FDA approval and comparability between patients and research/diagnostic labs. For colorectal cancer, 2–3 CTCs seem to be a feasible detection rate and serve as a method to predict a variety of clinical outcomes (reviewed in [138]). In a clinical study on locally advanced pancreatic cancer patients, ≥1 CTC predicted poor tumor differentiation, and worse overall survival [139]. In the context of a locally advanced and by definition not (yet) metastatic disease, the discovery of even single CTCs not only serves as a predictor but underlines the difficulty of detecting early metastatic disease and might even serve to support the treating physician in the choice between local vs. systemic therapy.

Interestingly, a study in pancreatic cancer demonstrated the usefulness of CTC quantification not only for prediction of prognosis but also for staging of the disease: Ankeny et al. were able to show that ≥3 CTCs in only 4 ml of whole blood discriminated between localized and metastatic disease [140].

Thus, while the individual cutoffs may vary between different studies, tumor stages, and entities, the detection of CTCs is now generally accepted as an important prognostic factor, and is increasingly used for disease staging, interim analyses and as a therapy response parameter in clinical studies. 

EMT and EMT-TFs have been closely associated with CTC generation [141,142]. Interestingly, YFP positive CTCs in a mouse model of skin squamous carcinoma were shown to have undergone EMT, implying that a partial EMT program is associated with an increased metastatic capacity [110]. KPC-YFP mice, the equivalent model in PDAC, allowed the identification of CTCs in 8–10-week-old mice bearing PanINs, revealing that the metastatic process occurs prior to full tumor development. These cells express different epithelial and mesenchymal markers, including Zeb1 [118]. These results were corroborated by single-CTC analyses from PDAC patients, which display variable gene expression profiles, allowing the establishment of epithelial-like or mesenchymal-like Zeb1 positive cells [143]. Interestingly, CTCs traveling in clusters have also been observed in the bloodstream of metastatic cancer patients [144]. Furthermore, in many tumors, clusters of migratory cells have been observed that retain cell-cell contacts and mesenchymal features, and display a higher metastatic potential than single migratory cells [145,146,147,148]. CTC clusters display stem cell characteristics, and binding sites for OCT4, Nanog and Sox2 have been found to be hypomethylated in these clusters, reminiscent of features seen in embryonic stem cells [149]. In line with this observation, single-CTC RNA-seq analysis of KPC tumor-bearing mice demonstrated loss of E-cadherin, whereas mesenchymal transcripts were more variable in their expression [150]. In another recent study, delaminating and invading single tumor cells that had undergone EMT were shown to lack E-cadherin expression while clusters of CTCs retained epithelial characters [151]. However, CTC clusters are rare in the peripheral blood and may get trapped in the capillary system. Moreover, when detected they appear to be associated with neutrophils, and predict shorter progression-free survival due to their higher metastatic potential [152]. In line with these works, hysteresis, or bistability of cellular states, drives TGF-beta—Zeb1 induced EMT in most normal and tumor mammary epithelial cells, as reflected by the bimodal distribution of high and low E-cadherin expression levels of cells exposed to TGF-beta, even after transient exposure, resulting in a more efficient tumor initiation and metastasis induction [153,154].

Although the association of EMT with CTCs and metastasis has been extensively endorsed, the position of EMT in the metastatic cascade is still under debate. Based on the use of different reporter lines and animal models, several works have claimed that EMT is dispensable for metastasis. Using fibroblast specific protein 1 (Fsp 1) to drive the conversion of RFP to GFP positive cells in primary breast tumor-bearing mice, lung metastases were found to be RFP positive and of epithelial origin, indicating that tumor cells did not activate Fsp1 to colonize the lung [155]. The generation of KPC pancreatic cancer mice with Twist or Snai1 deletion revealed that the capacity to form tumor spheres was unaffected by the lack of Twist or Snai1, and that these TFs were dispensable for metastases formation since the incidence of metastasis was similar to control KPC counterparts [156]. It is important to note that the elimination of Twist or Snai1 did not affect the expression of other EMT TFs in the aforementioned study, thus the possibility exists that Twist or Snai1 are not necessary or that other EMT TFs can compensate in their absence. Furthermore, studies in KPC animals containing a dual reporter system consisting of epithelial Pdx1 positive EGFP cells convertible into tdtomato-labeled cells under the control of Fsp1 or aSMA, showed that the majority of macrometastases are derived from epithelial (EGFP) cells. The authors, therefore, concluded that the establishment of metastases does not require an EMT program [157]. 

While such studies leave many questions unanswered, they raise the issue that not all TFs may be equally potent inducers of EMT, which might require a more clearly defined expression of the TF code to activate a partial program depending on the cellular context [158,159,160]. Additionally, the shortcomings of the approach using Fsp1 and aSMA have been recognized, since aSMA may be expressed in stromal cells and not acquired by neoplastic cells [161] and only a fraction of delaminated cells indeed express Fsp1 [118,125]. These results are consistent with cells which have undergone a partial EMT program being more efficient in disseminating and reverting to an epithelial state during the colonization process. Using the KPCY model, YFP+ cells were sorted according to their E-cadherin expression, defining two distinct clusters based on RNA-seq results: “complete EMT” with no E-cadherin expression, and “partial EMT” in the case of cells retaining E-cadherin transcripts, making an impact on the migration of the CTCs as single cells or clusters, respectively [162]. Interestingly, the authors demonstrate that E-cadherin repression is not transcriptionally mediated but rather the result of protein internalization, adding yet another layer of complexity to the understanding of EMT. Moreover, the authors found no evidence that the two programs coexist within the same tumor, suggesting that a partial EMT might represent the final manifestation of this process, and illustrates the variability in tumor behavior even within the same genetic background [162]. 

It is also worth noting the different expression patterns of EMT inducers in PDAC: Expression of Zeb1, Zeb2, Twist and Snai1 has clearly been detected in PDAC samples from human patients, where nuclear Snai1 and Twist expression are mostly observed in the stroma. The expression of Zeb transcription factors, however, was observed in both stromal and budding tumor cells [163], and similar results have been obtained in KPC mice [118]. The relevance of Zeb1 in particular in metastasis is undeniable since KPC-Zeb1 knock-out cell lines injected intravenously could not colonize the lung [120]. KC or KPC-derived GEMMs rely on the function of a recombinase usually driven by Pdx1 or Ptf1a. As mentioned above, Pdx1 is expressed during embryogenesis, so it could be plausible that deletion of a particular EMT-TF during development (although initially with non-redundant functions [164]) is compensated by another. Furthermore, EMT-TFs may exert specific functions in the progression from ADM through PDAC to metastasis, and their role might be pre-defined by the cells they are expressed in under physiological conditions in the first place (summary of EMT TFs and their individual roles in Table 1). As an example, Snai1 is expressed in mesenchymal cells in the normal adult pancreas. Therefore, Snai1 obliteration upon tamoxifen administration in normal acinar cells (under the control of a Ptf1a-Cre promoter) did not result in any abnormalities, as expected from a transcription factor exerting cell-autonomous functions [105]. On the other hand, Snai1 overexpression in elastase positive acinar cells did not affect tissue morphology or the response to cerulein-induced pancreatitis [165]. In PDAC, as mentioned before, Snai1 maintains its expression most prominently in the stroma. Among the stromal components, CAFs are a heterogeneous cell population of mesenchymal origin highly abundant in carcinomas, and PDAC in particular, where PSCs seem to be their major source [166]. Messal et al. have developed a new technique for whole organ 3D imaging (FLASH), enabling the visualization of pancreatic duct oncogenic transformation originating from two types of lesions: Exophytic, expanding basally from small ducts, and endophytic, expanding into the lumen of larger ducts. Exophytic lesions displayed more prominent EMT and were more efficient in recruiting CAFs, contributing to the aggressiveness of the lesion [167]. CAFs, in turn, stimulate the invasion of cancer cells via direct contact with tumor cells, leading a thin column of delaminating tumor cells that follow behind [168,169]. Reciprocal juxtacrine and paracrine signaling between CAFs/stromal cells and cancer cells also impact on the development and metastasis of PDAC [170,171]. Indeed, co-cultures of different ratios of pancreatic cells with CAFs revealed their mutual influence promoting shifts to inflammatory CAFs, and to cancer cells with double proliferative and EMT phenotypes, resulting in increased ability of individual cancer cells to proliferate and metastasize [172].

Disseminated cells must adapt to infiltrate the new environment, which further limits metastatic colonization. For migratory cells to seed distant organs, not only does the idiosyncrasy of the migrating CTCs determine the final destination, but organotropism to the recipient tissue is an additional prerequisite [173]. Soluble serum factors known to prime the metastatic niche in the liver are exosomes and tissue inhibitor of metalloproteinases-1 (TIMP1). SDF-1 in the metastatic niche, in turn, recruits a subpopulation of CD133/CXCR4-expressing CSCs [37]. Stromal cells and in particular CAFs, signaling through their secretome, in particular through the action of LIF, activate IL-6-STAT3 signaling in both pancreatic cancer cells and hepatocytes which then produce the chemoattractant SAA to establish a pro-metastatic niche [161,174,175]. Interestingly, in the context of OSKM (Oct4, Sox2, KLF, cMYC) overexpressing mice, IL-6 was identified as a critical factor secreted by senescent cells to drive stemness and EMT [176,177]. Furthermore, TFs associated with EMT, such as Twist1/2, ZEB1/2, Snai1/2, and YAP can confer senescence escape mechanisms to certain cells [178,179,180].

## 7. EMT and Therapy Resistance

A key issue in therapy resistance are pre-existing (or therapy-induced) chemoresistant clones, which appear to be an inherent feature of cancer cell subpopulations, concurring with drug-induced plasticity. For example, Seth et al. uncovered alterations in the clonal composition of relapsed tumors following chemotherapy administration, and defined functionally heterogeneous subpopulations with different drug sensitivity [92]. Moreover, drug-resistance in PDAC cell cultures has been shown to promote the selection of preexisting clones in tumors of heterogeneous origin, whereas homogenous tumors display drug-induced plasticity through the emergence of trans-differentiated cells. The underlying mechanism of this drug-induced adaptation process resides in loss of the stem cell factor Sox2 and subsequent gain in Sox9 on drug-induced H3K27ac sites, suggesting that adaptation and TICs could also be driven by epigenetic plasticity [185]. Indeed, Sox9 was identified as a transcription factor enriched in stem cells by single-cell transcriptomic and epigenetic analysis, which revealed the uniqueness of H3K27ac super-enhancers to either stem or non-stem cells [60].

It is widely accepted that therapy resistance is one of the major mediators of tumor relapse. As a result, chemotherapy depletes the bulk of the tumor but increases the number of drug-resistant CSCs, including CD133-positive and [37] ALDH-positive cells [186]. Notable CSC features found to be driving resistance are dormancy/quiescence, increased DNA repair [187] and levels of efflux transporters, upregulation of antiapoptotic proteins, epigenetic regulation [188] and factors from the TME [189,190]. The chemoresistance of CSCs relies on the activation of several key signaling pathways such as Hedgehog, mTOR, Nodal/Activin, NF-kB, Notch, TGF-beta or Wnt/b-catenin signaling pathways, many of which are also important for the regulation of stem and progenitor cells as well as for normal embryonic pancreas development [191,192]. Indeed, combined inhibition of several signaling pathways together with gemcitabine treatment has been shown to be efficient in depleting the CSC population and to result in long-term survival in mice [76,187,193,194]. As a confirmation of the potential efficacy of CSC-targeted treatment, clinical studies have demonstrated significant benefits in colorectal and pancreatic cancer with combination therapy using the STAT3 inhibitors napabucasin plus chemotherapy (NCT02231723, NCT02993731). 

The tumor microenvironment contributes to hypoxia, and oxygen deprivation stabilizes HIF1a, which has been involved in drug resistance via different mechanisms [195]. PSCs activate the CSC marker c-Met through secretion of its specific ligand, hepatocyte growth factor, conferring therapy resistance [196]. Stromal immune cells also contribute to CSC maintenance and chemoresistance: Targeting TAMs by inhibiting colony-stimulating factor-1 receptor (CSF1R) reduced the number of CSCs and improved therapeutic efficacy [197]. Even intratumoral bacteria have been shown to contribute to gemcitabine resistance by metabolizing the drug and reducing its concentration within the tumor [198].

EMT-TFs can also mediate reduced proliferation and resistance to cell death (even when induced by radio or chemotherapy) [199,200,201]. In cell culture, chemoresistant subclones analyzed for migration and invasion properties led to the identification of EMT-TFs linked to drug-induced epithelial-mesenchymal plasticity [181,184]. The mechanisms by which EMT-TFs confer resistance seems to be via the regulation of drug-inactivating enzymes and drug transporters: KPC mice lacking Twist or Snai1 were sensitive to gemcitabine concomitantly with upregulation of the gemcitabine transporters ENT1 and CNT3 [156]. Cells which acquired expression of EMT-TFs also showed upregulation of drug-metabolizing enzymes and efflux transporters involved in multiple drug resistance [155,202]. Furthermore, exosomes released from gemcitabine-treated cells confer chemoresistance through the upregulation of ROS detoxifying enzymes and the downregulation of the gemcitabine metabolizing enzyme DCK [203], and exosomes released by CAFs treated with gemcitabine stimulated Snai1 expression in tumor cells, promoting therapy resistance [204]. EMT-TFs also limit DNA damage to maintain chemoresistance themselves [182].

Oncogene activation induces DNA damage and chromosomal instability, and the subsequent cellular DNA damage response promotes the activation of the cell cycle checkpoint kinases ATM ad ATR to either maintain genome integrity, or enter into cell cycle arrest or apoptosis, depending on the extent of the DNA damage. EMT-TFs also limit DNA damage to maintain chemoresistance via different mechanisms: ATM regulates Snail stabilization, resulting in increased metastasis and invasion of breast cancer cells [205]. Zeb1, once stabilized by ATM, contributes to CHK1 expression and subsequent radioresistance [206]. H2AX, a histone H2A variant involved in DNA repair, was shown to directly bind and repress the Snai2 and Zeb1 promoters in colon cancer cells. Subsequently, H2AX depletion induced EMT, making these cells more prone to invasion [207]. ZNF281, a zinc-finger transcription factor implicated in EMT induction through a feed-back loop with Snai1, is activated by DNA damage-inducing drugs in different cancer cell models, and directly regulates XRCC2 and promotes DNA repair [208]. In PDAC, Prrx1 interacts with FoxM1 to limit DNA damage induction [182], and when PDAC cells were stratified based on their KRAS dependency, YAP and Zeb1 were identified as KRAS-independent factors which promote cell cycle and DNA replication, and survival together with EMT features, respectively [183,209].

Several important EMT-TFs are differentially expressed during carcinogenesis and tumor progression, varying even between different stages, and with non-redundant functions [164]. The pleiotropic action of EMT-TFs and the tight association between stemness and EMT, but also therapy resistance (including immunotherapy) and immune suppression, make these TFs very interesting targets for the development of personalized tumor therapies.

Clinical studies specifically dedicated to investigate the effects of anti-stemness or anti-EMT drugs are still scarce. In addition to the promising napabucasin trials mentioned above, other clinical trials have been started with variable effects. While metformin treatment was effective in preclinical models to eliminate oncogenic progression and CSCs [83,130], a recent clinical trial showed no benefit to overall survival with metformin added to gemcitabine and erlotinib treatment of locally advanced pancreatic cancer [210]. Inhibition of mTOR in patient-derived pancreatic cancer xenografts was comparable to patient response to mTOR inhibitors, but surprisingly could not be predicted by screening for pathway activation [211].

Approaches using EMT inhibitors for PDAC clinical treatment are still limited, and only a few studies evaluate the direct effects of EMT-related targets such as E-Cadherin, N-Cadherin, and vimentin (NCT02913859) or secreted clusterin (NCT02412462). Nonetheless, the use of an EMT signature or intermediate states of EMT in CTCs is becoming an important part of many clinical studies in solid tumors. Furthermore, a plethora of new targets and agents are currently under intense investigation, demonstrating the potential of EMT-related treatment (compiled in Table 2).

## 8. Future Directions

The intricate interplay between stemness and EMT and the mutual regulation of these two seemingly distinct processes is the subject of intense investigation. Given the heterogeneity of PDAC and CSCs, as previously discussed, it is also worth noting that differences in gene mutations and molecular pathways could induce particular EMT programs or transcription factors, concomitant with different downstream mechanisms and targets. Therefore, many of the players involved may still need to be identified and the individual role of each regulator still needs to be further dissected. 

However, data from early clinical trials clearly point to the disruption of stemness and/or EMT in pancreatic (and other) cancers as a promising approach that could revolutionize patient therapy. Strategies to tackle therapy resistance or an increase in the susceptibility of CSCs to conventional therapeutics could include inhibition of EMT induction and/or target the mesenchymal invasive phenotype. However, the precise roles of these compounds need to be better understood, as suggested by negative clinical study data, and their use might be ill-advised in patients with late-stage PDAC: In these situations, tumor cells will already have undergone EMT, and these strategies might promote MET or cell epithelialization and might do more harm than good. We envision that the combination of different approaches, including analysis of patient-specific mutations and EMT stage to define combined, personalized therapies, would be more clinically efficient. Despite the advances made in systemic therapies, immunotherapy and nanoparticle-based systems show great potential to improve drug delivery and overcome the aforementioned issues by targeting specific organs and/or cells with increased efficiency (e.g., targeting of senescent cells) [232]. Furthermore, new biomarkers able to quantify the therapeutic effects of new drugs may need to be considered, since gross reduction of tumor size or overall survival may not be the appropriate parameter for (transiently) successful treatment. Therefore, the discovery of further links between these processes and of new (targetable) players in this network deserves our full attention. To this end, a better understanding of the data generated in genetically engineered mouse models translated into a clinical setting will be of paramount importance.

## 9. Conclusions

While the role of CSCs in (pancreatic) cancer biology is yet to be fully understood, the extensive research published since their discovery in 2007 has provided a tremendous amount of knowledge regarding not only their identification but, even more importantly, their role in tumor maintenance, relapse, and metastasis. The essential role that EMT plays in various processes leading up to and during metastatic dissemination has been appreciated for a long time. However, that EMT and stemness may be intricately linked and even jointly regulated in cancer (stem) cells is new and opens up a new horizon for understanding two seemingly different features in cancer at the same time. The further study of the molecular mechanisms behind PDAC progression and drug resistance, including the detailed analysis of the role of EMT-TFs, will undoubtedly lead to the identification of novel targets and prognostic markers for more effective therapeutic targeting.

## Figures and Tables

**Table 1:** Key EMT regulators.

EMT Regulators in Embryonic Development of the Pancreas			
Gene	Assay	Description	Ref.
*Snai1*	expression pattern	in situ hybridization	[99]
	expression	single cell RNAseq	[101]
*Snai2*	expression pattern	in situ hybridization, immunofluorescence	[99,102]
	expression	single cell RNAseq	[101]
	functional analysis	Snail2 electroporation promotes cell delamination	[99]
*Prrx1*	expression	microarray	[100]
*Twist*	expression	single cell RNAseq	[101]
*YAP*	expression pattern	Immunofluorescence	[104]
	functional analysis	Conditional KO shows reduction in endocrine cells	[104]
*E-cadherin/Vimentin*	expression	Coexpression in Ngn3+ cells shown by single-cell PCR	[98]
EMT Regulators in PDAC progression			
Gene	Assay	Description	Ref.
*Snai1*	Conditional KO in KPC mice	Dispensable for metastasis, promotes chemoresistance	[156]
	Expression by qPCR, WB	Upregulation of Snai1 in Panc1 spheres enriches for stemness markers	[122]
*Snai2*	Cd133 KD in Capan1 M9 cells	Stemness marker CD133 regulates Snail2 expression	[121]
*Twist*	conditional KO in KPC mice	Dispensable for metastasis, promotes chemoresistance	[156]
*Zeb1*	expression in KPCY mice	Present in CTCs	[118]
	KD in human cells	Promotes chemoresistance	[181]
	Conditional KO in KPC mice	Critical for stemness and metastasis	[120]
*Prrx1*	OE and KD	Differential isoform regulation of Sox9-mediated stemness	[100]
	Inducible OE in KPC mice	Isoform switching regulates EMT states (delamination & metastasis)	[123]
	OE in human cells	Limits DNA damage	[182]
*Fsp1/aSMA*	Lineage tracing in KPC mice	Do not contribute to metastasis	[157]
*YAP*	KD in human cells	YAP1 amplification can promote KRas independent recurrence	[183]
EMT signature	Notch KD in human cells	Notch signaling promotes EMT-mediated chemoresistance	[184]
	Patient CTCs	Single-cell qPCR showed enrichment in mesenchymal markers	[143]
	KCYp120ctnwt/+ mice	Mono-allelic p120ctn loss shifts metastatic burden to the lung	[151]
	KPCY mice	Tumor cells retain E-cadherin transcripts during EMT	[162]
	KPC mouse single-cell RNAseq	Stem cells and mesenchymal signatures show overlap	[60]
	Patient-derived cells	EMT signature contributes to metastasis and chemoresistance	[172]

KO = Knockout, KD = Knockdown, OE = Overexpression, WB = Western blot.

**Table 2:** Drugs/approaches targeting EMT.

Drugs Used for Targeting EMT in PDAC				
Type of Drug	Compound	Target	Description	Ref.
Epigenetic	Mocetinostat	HDAC	HDAC I inhibitor restores miR-203 expression to downregulate Zeb1	[212]
Antibiotic	Salinomycin	RhoA	Loss of actin stress fibers and reduced metastasis	[213]
Anti-malaria	Chloroquine	Autophagy	Blockade of autophagy	[214,215]
		EMT	CXCR4 and hedgehog signaling inhibition with subsequent EMT inhibition	[216]
Anti-diabetic	Metformin	OXPHOS	Inhibition of CSCs by Gata6 upregulation and decrease of Snail1	[130]
Metabolism	Glycolytic and Glutaminolytic Inhibitors	EMT signature	Correlation of EMT signature with sensitivity to specific inhibitors	[217]
Natural compound	Withaferin-A	Nestin	Suppression of metastasis	[218]
	Triptolide	NF-kB	Inhibition of hypoxia and Twist2 induced stem-like features	[219]
Monoclonal antibody	Tarextumab	Notch2/3	Reversed *Snail* and *Twist* upregulation mediated by Gemcitabine	[220]
Nanoparticles	Mangostin	SHH	Downregulation of Snail, Slug, Zeb1 and N-cadherin	[221]
Small molecule inhibitors	Apricoxib	COX2	Reverses EMT	[222]
	Erlotinib	EGFR	Suppresses cancer metastasis	[223]
	cyclopamine	SHH	Inhibits Snai1 mediated EMT	[224]
	LY2109761	TGFbRI/II	Suppresses cancer metastasis	[225]
	SB-431542	TRKI	Attenuates TGF-beta-induced EMT	[226]
	VS-4718	FAK	Reduction of ALDH and CD44 together with metastasis	[227]
	Stattic	STAT3	Reduced migration and invasion	[228]
	LY294002	PI3K/Akt	Decreased expression of vimentin, Snail1 and Snail2	[229]
	PD0325901	MEK	Attenuates TGF-beta-induced EMT	[230]
	Neratinib	ERBB1/2/4	Translocation of YAP to the cytosol	[231]
